# Supplementary material for: Beneficial effects of bempedoic acid treatment in polycystic kidney disease cells and mice
Source: Front Mol Biosci. 2022 Nov 25;9:1001941. doi: 10.3389/fmolb.2022.1001941 (PMC9730828; doi:10.3389/fmolb.2022.1001941)
Supplement: Supplementary file 1 [file DataSheet2.PDF]

SUPPLEMENTARY FIGURE LEGENDS

Supplementary Figure 1 – The bempedoic acid-activating enzyme ACSVL1 (FATP2) is expressed in IMCD-derived *Pkd1$^{-/-}$* kidney cells. A. $^{-}$Both FATP2a and FTAP2b isoforms are expressed in this immortalized mouse kidney epithelial cell line of collecting duct origin by immunoblot. FATP2a expression in *Pkd1* KO cells (ID1-3E5, *Upper, right*) than in controls (WT IMCD3, *Upper, left*). *Lower*, total protein signal as loading control for the immunoblot. **B.** Densitometric quantification of the FATP2a levels normalized to total protein indicates that the *Pkd1*-null IMCD-derived cell line (*Pkd1* KO) express approximately 40% less FATP2a than the wild-type IMCD3 cells (WT). Three representative lysate immunoblots are shown for each condition (***P* < 0.01).

Supplementary Figure 2 – ACLY inhibitors dramatically inhibit cystic growth in 3D cultures in IMCD-derived *Pkd1$^{-/-}$* kidney cells. Bempedoic acid (BA) and SB-204990 inhibit cyst growth of IMCD-derived *Pkd1$^{-/-}$* (ID1-3E5) kidney epithelial cells in 3D cultures. Cells were grown in Matrigel for a total of 7 days, supplemented with forskolin + IBMX after day 1, and then treated with either vehicle (DMSO), BA or SB-204990 for the last 3 days. **A.** Representative images of cystic structures under the different treatment conditions (scale bar = 100 μm). **B.** Summary data reveal that treatment with either BA or SB-204990 significantly reduced cyst area relative to CON (data from one experiment, n = 21-28, *****P* < 0.0001 for the indicated comparisons).

Supplementary Figure 3 – Bempedoic acid inhibits mitochondrial superoxide production and promotes mitochondrial elongation in IMCD-derived *Pkd1$^{-/-}$* kidney cells. A. To analyze the effect of BA on mitochondrial superoxide production, IMCD-derived *Pkd1$^{-/-}$* (ID1-3E5) cells were stained with MitoSOX™ Red mitochondrial superoxide indicator (*red*) and Hoechst 33342 nuclear stain (*blue*). **i.** Representative epifluorescence micrograph images are shown of ID1-3E5 cells in the absence (*top*) or presence (*bottom*) of BA treatment (100 μM) for 24 h. **ii.** Summary data reveal that BA treatment dramatically decreased mitochondrial superoxide production in ID1-3E5 cells (data from one experiment, n = 26-31 cells analyzed; *****P* < 0.0001). **B.** BA treatment significantly increased mitochondrial elongation of *Pkd1*-null cells. **i.** Representative images of MitoTracker Deep Red-stained ID1-3E5 cells in the absence (*top*) or presence (*bottom*) of BA treatment (100 μM) for 24 h. **ii.** Summary data reveal that BA treatment significantly increased mean cellular mitochondrial elongation in ID1-3E5 cells (mean cellular mitochondrial elongation values from one experiment with n = 26-28 cells analyzed, as described in *Materials and Methods*; **P* < 0.05).

Supplementary Figure 4 – Effects of bempedoic acid and tolvaptan treatment in PKD mice on protein expression of the bempedoic acid-activating enzyme ACSVL1 (FATP2). A. *Upper*, immunoblot of kidney tissue homogenates from *Pkd1$^{fl/fl}$;Pax8-rtTA;Tet-O-Cre* mice with doxycycline-induced *Pkd1* gene inactivation with or without concurrent treatment with BA (30 mg/kg/d) and/or tolvaptan (30 or 100 mg/kg/d) for FATP2a. *Lower*, total protein signal as loading control for the immunoblot. **B.** Densitometric quantifications of the FATP2a levels normalized to total protein indicate significant increases in BA- and tolvaptan-treated animals compared to vehicle control. (**P* < 0.05, ***P* < 0.01 for the indicated comparisons).
