# Supplementary material for: Beneficial effects of bempedoic acid treatment in polycystic kidney disease cells and mice
Source: Front Mol Biosci. 2022 Nov 25;9:1001941. doi: 10.3389/fmolb.2022.1001941 (PMC9730828; doi:10.3389/fmolb.2022.1001941)

Supplementary Figure 1

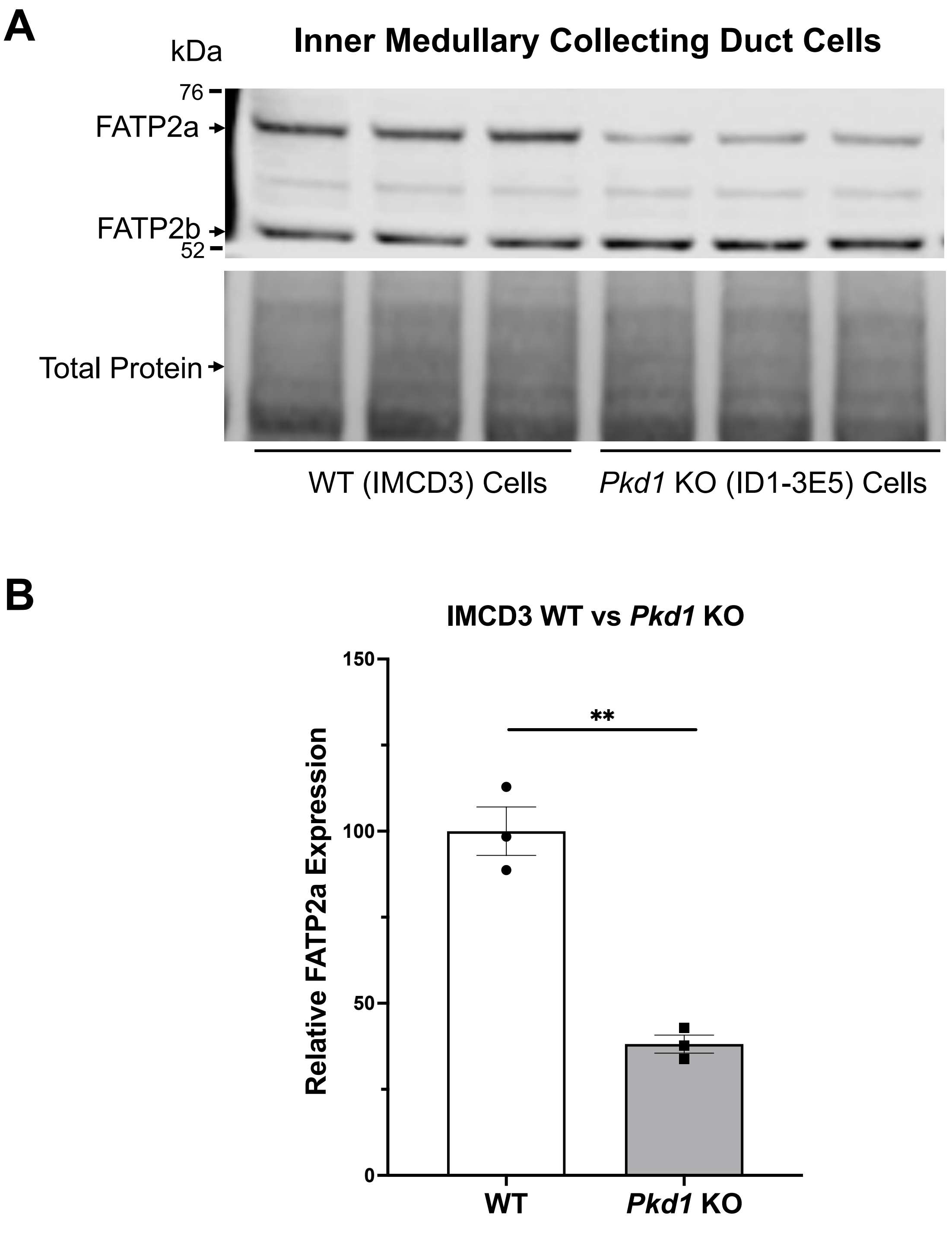

Supplementary Figure 2

A

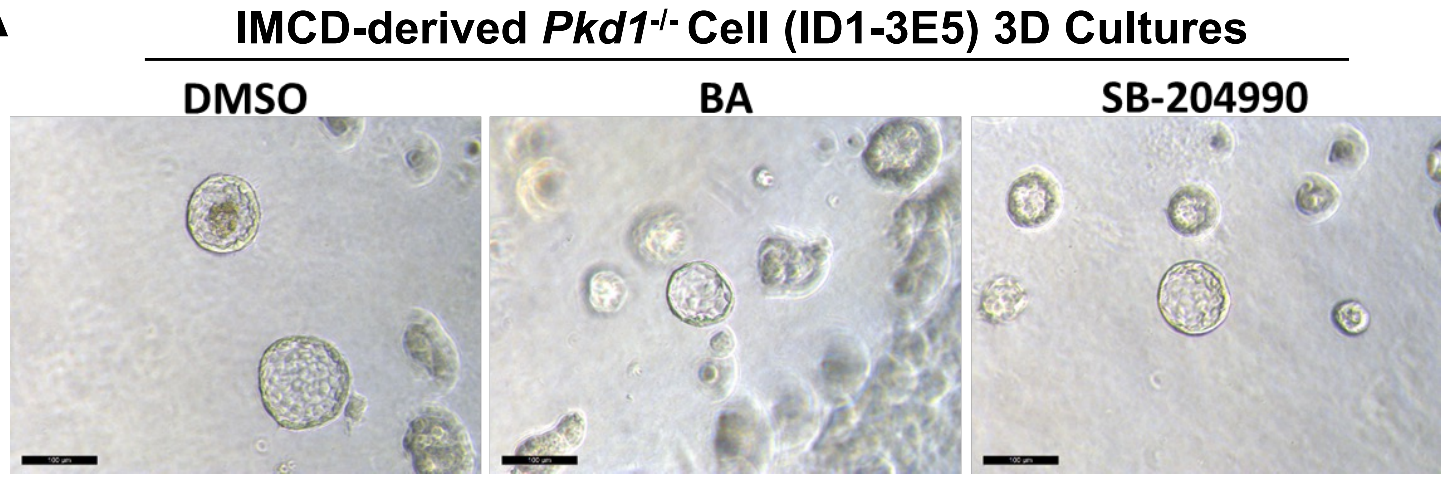

B

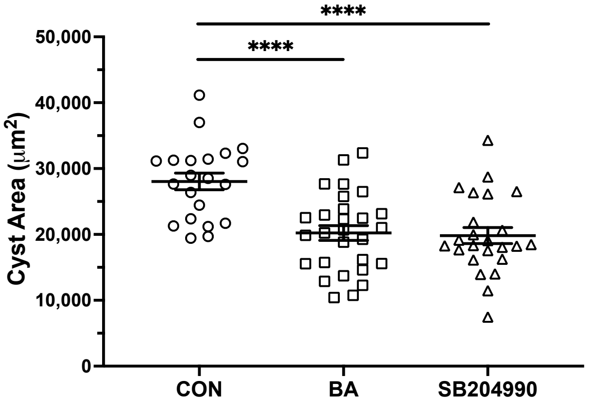

Supplementary Figure 3

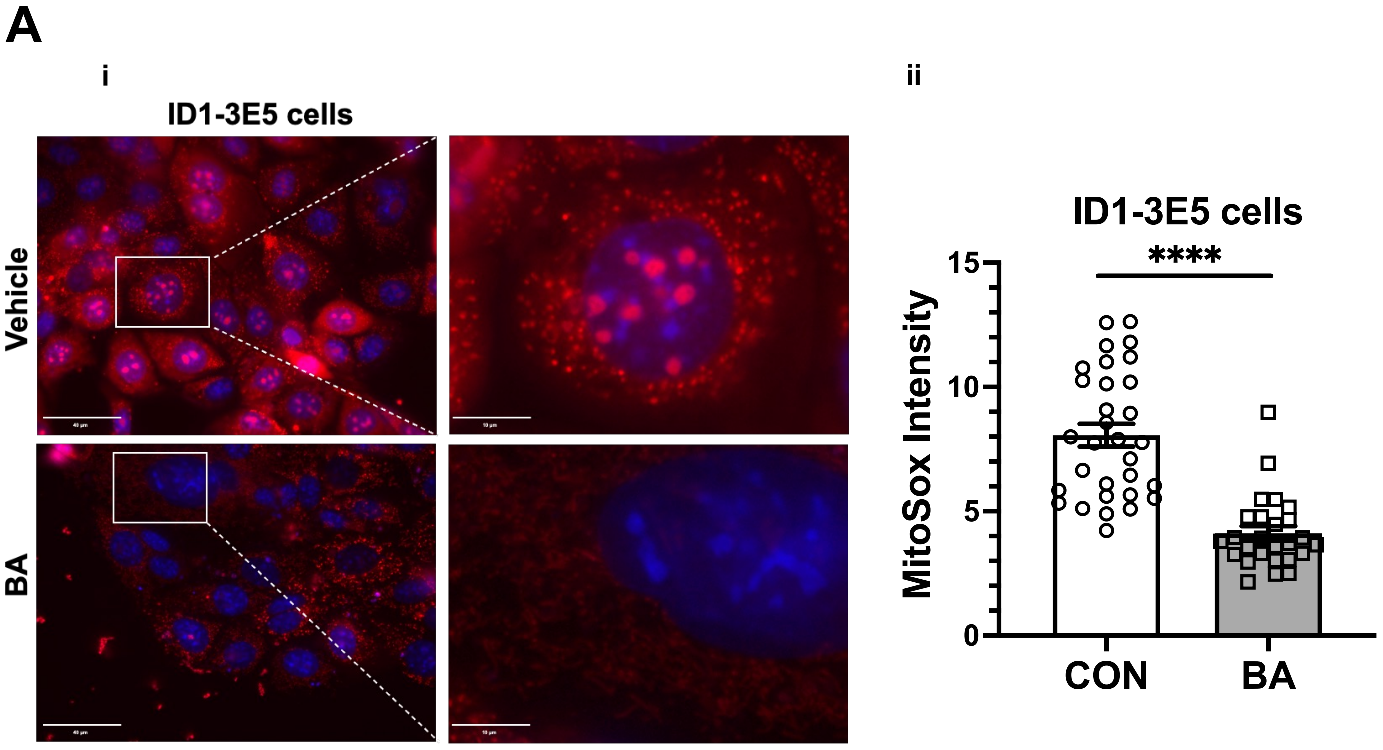

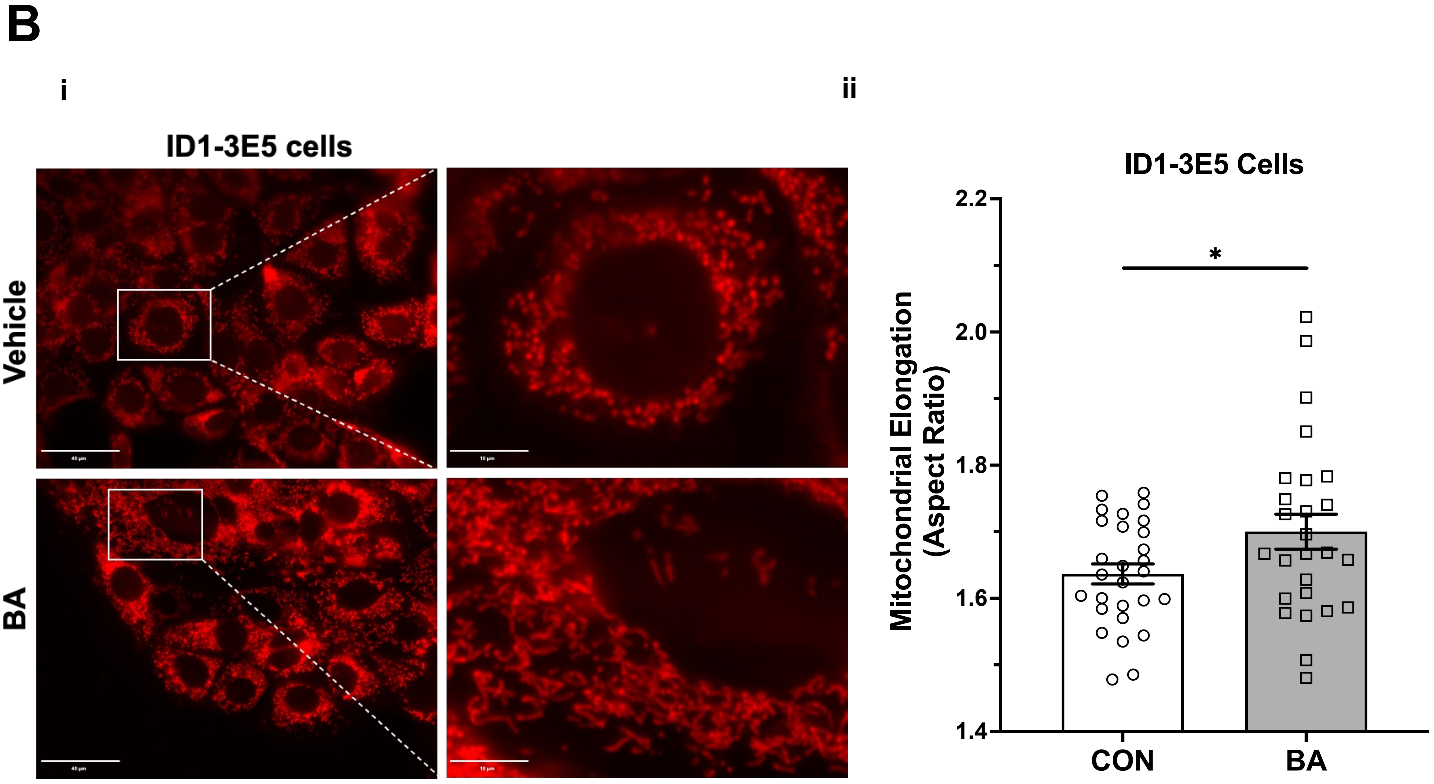

Supplementary Figure 4

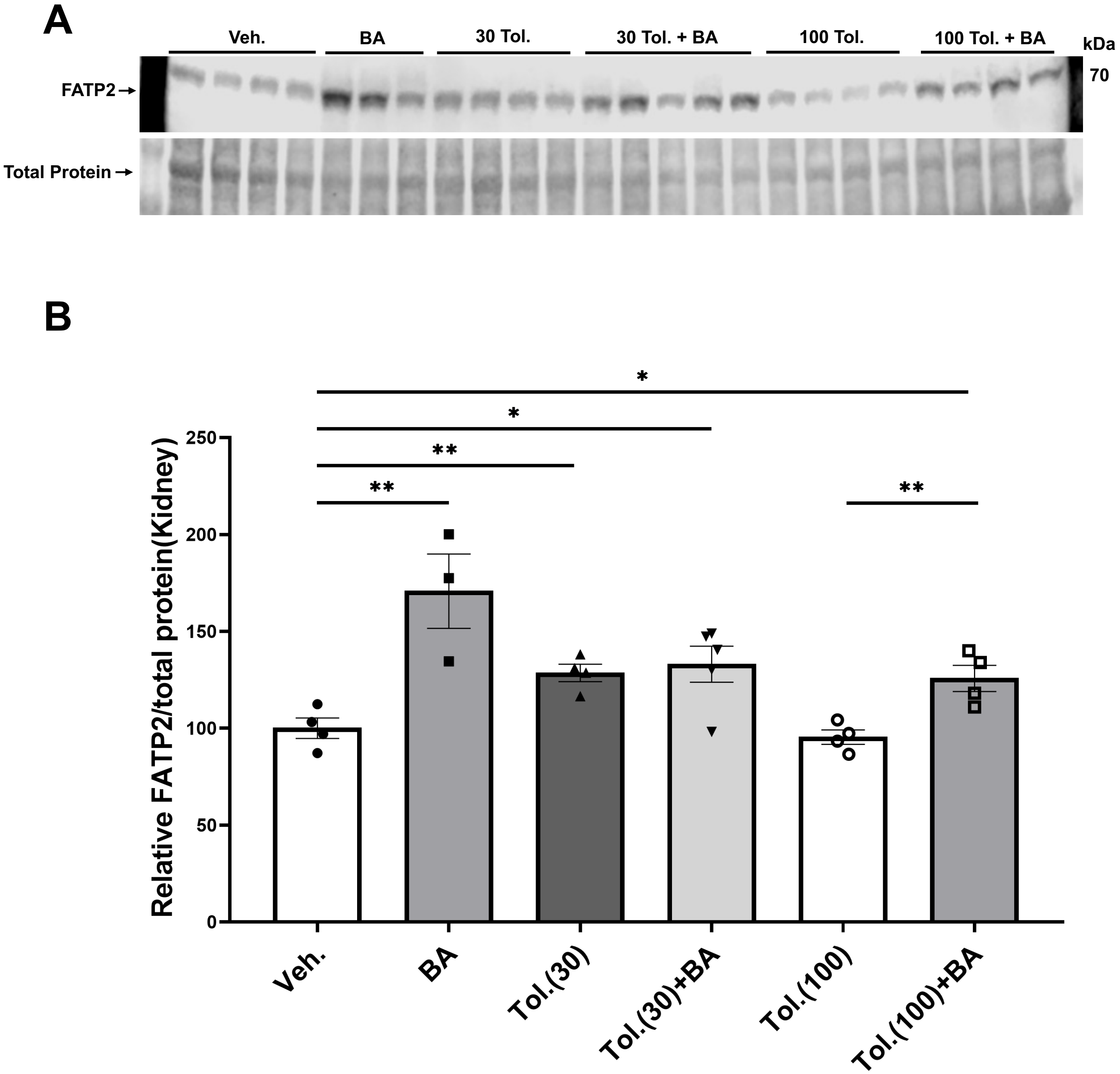

Supplement: Supplementary file 2 [file DataSheet1.PDF]
